# Reproductive defects in the abscission mutant *ida-2* are caused by T-DNA–induced genomic rearrangements

**DOI:** 10.1093/plphys/kiad449

**Published:** 2023-08-09

**Authors:** Renate Alling, Sergio Galindo-Trigo

**Affiliations:** Section for Genetics and Evolutionary Biology, Department of Biosciences, University of Oslo, 0316 Oslo, Norway; Centre for Ecological and Evolutionary Synthesis, Department of Biosciences, University of Oslo, 0316 Oslo, Norway; Section for Genetics and Evolutionary Biology, Department of Biosciences, University of Oslo, 0316 Oslo, Norway

Dear Editor,

Our understanding on the molecular regulation of abscission, the developmentally programmed separation of plant organs, is largely owed to the discovery and characterization of a secreted peptide: INFLORESCENCE DEFICIENT IN ABSCISSION (IDA) (Butenko et al. 2003). In Arabidopsis, IDA is secreted in the abscission zones of floral organs and in root cortex cells overlaying the site of lateral root emergence (Kumpf et al. 2013). In these tissues, IDA is perceived by plasma membrane receptor kinases to promote cell separation, facilitating floral organ shedding and lateral root emergence, respectively (Jinn et al. 2000; Cho et al. 2008). Unraveling how IDA is perceived by its receptors has not only informed the abscission field but has also served as a model system to understand the mechanistic basis for small peptide perception by receptor kinases (Meng et al. 2016; Santiago et al. 2016; Hohmann et al. 2018). Since 2008, the *ida-2* line has become the most widely used *ida* allele due to its convenient Col-0 genetic background (Cho et al. 2008). *ida-2* has been used in numerous studies to identify new abscission regulators or decipher how different stresses influence abscission (Shi et al. 2011; Kumpf et al. 2013; Liu et al. 2013; Patharkar and Walker 2016; Patharkar et al. 2017; Lee et al. 2018; Zhu et al. 2019; Roman et al. 2022). Importantly, the *ida-2* mutant is recurrently used in studies where the knowledge generated in Arabidopsis is applied to plants of agricultural or ornamental interest (Estornell et al. 2015; Ying et al. 2016; Kim et al. 2018; Verma et al. 2022; Singh et al. 2023). We have found evidence for chromosomal translocations and additional uncharacterized T-DNA insertions in the *ida-2* line. These previously unnoticed genomic features have a deleterious impact on gametogenesis when *ida-2* plants are crossed to plants with a regular genomic architecture, resulting in substantial reproductive defects and genetic linkage between different chromosomes.

We first observed atypical segregation ratios in the F2 generation of a genetic cross between the *ida-2* line and *idl1cr1* line (*IDA*, *AT1G68765*, chromosome 1; *IDA-like1* [*IDL1*], *AT3G25655*, chromosome 3) (Shi et al. 2018). Three genotypes were overrepresented in the F2: double hemizygotes (*ida-2/IDA idl1cr1/IDL1*) and parentals (*IDA/IDA idl1cr1/idl1cr1* and *ida-2/ida-2 IDL1/IDL1*), whereas, interestingly, there was a complete absence of wild-type (WT) plants and double homozygous mutants (Supplemental Table S1). The absence of WT plants in the F2 generation suggested meiotic progression defects in the F1 plants of the *ida-2* x *idl1cr1* cross. We investigated the male and female gametophyte development in this cross and observed a gametophyte development impairment in plants hemizygous for both *ida-2* and *idl1cr1*, while plants carrying only 1 of the mutated genes were fully fertile (Supplemental Fig. S1).

Although we initially speculated the gametophytic impairment could be explained by a functional interaction between the *IDA* and *IDL1* genes, the same reproductive impairment was seen in the F1 generation of a cross between *ida-2* and WT, suggesting the impairment was solely associated with the *ida-2* line (Fig. 1, A and B; Supplemental Fig. S2). Interestingly, it was also noticed that previous efforts to obtain double mutants between *ida-2* and mutants in the genes *AT3G16555* and *IDL4* had also failed (not shown). *IDL1*, *IDL4*, and *AT3G16555* are located in proximity within chromosome 3 (chr3; 5633426-9338075 bp). Possible genetic linkage between the *ida-2* T-DNA insertion in chr1 and three independent genes located in proximity in chr3, together with the fertility defects in descendants of *ida-2* crossed to WT plants, suggested a T-DNA-induced chromosomal rearrangement between the *IDA* gene in chr1 and the region of chr3 containing *IDL1*, *AT3G16555*, and *IDL4* (Curtis et al. 2009).

**Figure 1.**
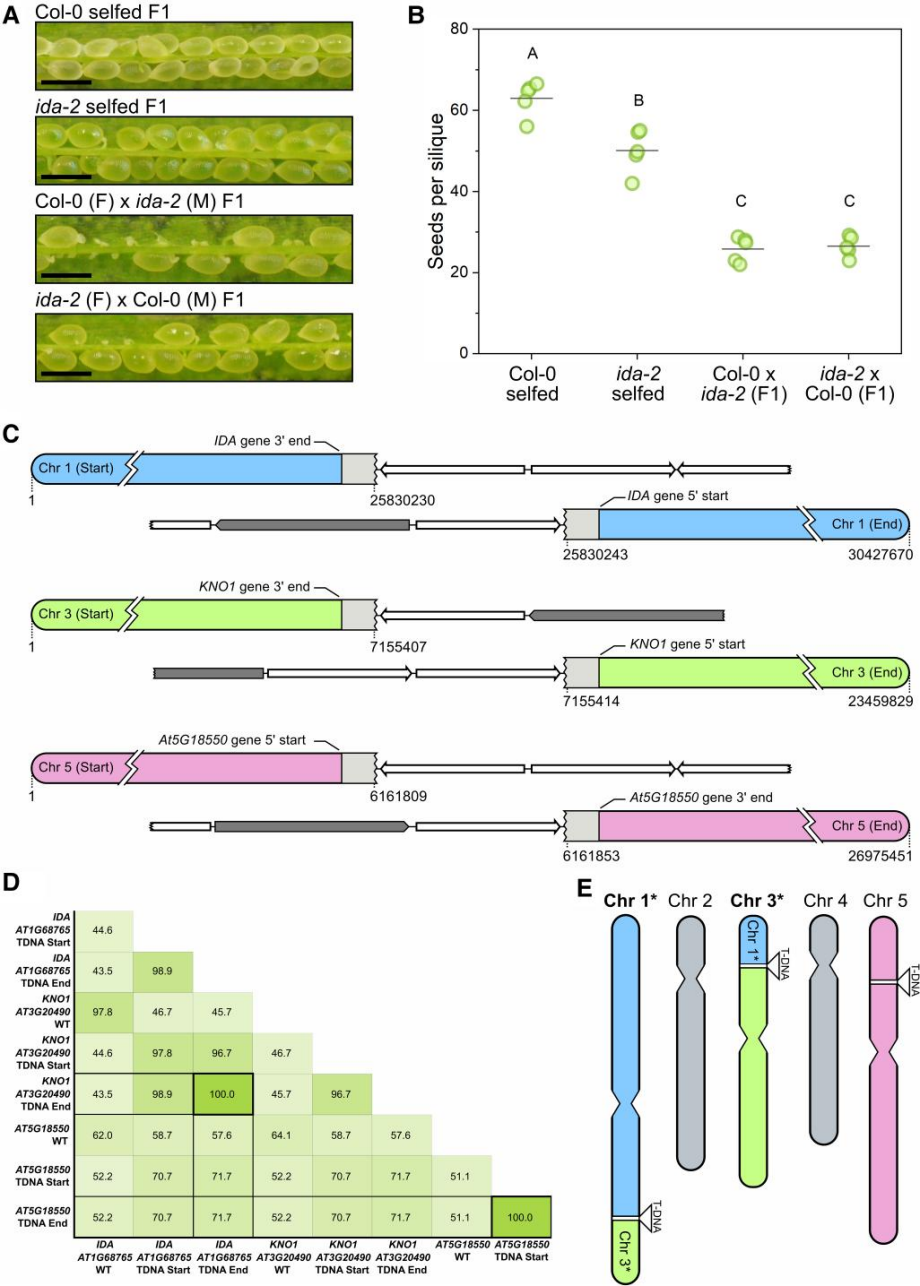
T-DNA-induced genomic structural variation explains the *ida-2* fertility defects and genetic linkage between the *ida-2* insertion and chromosome 3. **A)** Representative images of fertilized seeds growing in dissected siliques of F1 plants of the indicated crosses. Many unfertilized ovules can be seen in the F1 generation of crosses between *ida-2* and WT (Col-0) plants. Scale bars are 0.5 mm. **B)** Quantification of seed production per silique as a proxy for the *ida-2* fertility defects. Data correspond to the average seed per silique in 5 siliques per plant. Five independent plants per cross were analyzed. Different letters indicate statistically significant differences in a 1-way ANOVA with post hoc pairwise comparisons using Bonferroni and Tukey tests (*P* < 0.05). See also Supplemental Fig. S2. **C)** Diagram of the detected T-DNA insertions in the *ida-2* whole-genome sequencing analysis. Homozygous insertions were present in *IDA*, *KNO1*, and *AT5G18550*, although the read length (14 kb in average with PacBio HiFi) was insufficient to assemble the entire T-DNA. Arrows depict the *pROK2* T-DNA sequence, whereas the dark grey boxes represent the *pROK2* vector backbone. Dotted lines followed by a number indicate the nucleotide position based on the TAIR10 reference genome. **D)** The coincidence coefficient of the selected genomic features genotyped in 92 plants in the F2 population of the *ida-2* × *idl1cr1* cross shows the genetic linkage between the T-DNAs in chr1 and chr3 (*IDA* and *KNO1*, respectively). The T-DNA insertion in chr5 (*AT5G18550*) segregated independently from those in chr1 and chr3. **E)** Proposed karyotype in the *ida-2* line based on the reproductive phenotype, segregation analyses, and whole-genome sequencing data.

We sequenced the genome of the *ida-2* mutant and found, besides the *IDA* gene T-DNA insertion, homozygous T-DNA insertions in the gene *KNOTEN1* (*KNO1*, *AT3G20490*), found in the expected neighboring region of chr3 described above, as well as in the gene *AT5G18550* (Fig. 1C). We examined the segregation of both junctions of each of the 3 T-DNA insertions in 92 F2 plants in the *ida-2* x *idl1cr1* genetic cross. When independently analyzed, all genotyped loci appeared at the expected proportions in the 92 F2 plants (Supplemental Tables S2 and S3). However, pairwise cosegregation analysis of each of the 3 T-DNA insertions from the *ida-2* line indicated the genotyped loci in chr1 and chr3 were linked, while the T-DNA insertion in chr5 segregated independently from both chr1 and chr3 insertions (Supplemental Table S4). The coincidence coefficient between the examined genomic loci revealed a linkage between *IDA* and *KNO1* T-DNA insertions in chr1 and 3 (Fig. 1D). Based on this evidence, the *ida-2* karyotype is expected to be as depicted in Fig. 1E. Such genomic structure would explain the genetic and developmental phenotypes observed in outcrossed *ida-2* plants. Firstly, structural variation between genomes in an F1 hybrid can cause gametophyte abortion due to genome unbalance in meiocytes after recombination and reduced seed yield per silique as a result. Secondly, a translocation between the T-DNA insertion sites in *IDA* and *KNO1* would impair segregation between *IDA* and the genes neighboring *KNO1* in chr3.

Next, we generated an alternative *ida* mutant allele in the Col-0 accession. Using CRISPR-Cas9, we produced plants with a single nucleotide insertion in the 34th codon of *IDA* (Fig. 2A; Supplemental Fig. S3). This mutation results in a frameshift that impedes translation of the mature IDA peptide (Fig. 2A; Supplemental Fig. S3A). We named this new allele *idaCR*. Homozygous *idaCR* plants showed an indistinguishable floral organ abscission phenotype to *ida-2*, confirming the disruption of the active IDA peptide (Fig. 2, B and C; Supplemental Fig. S3B). When the *idaCR* line is crossed to WT, F1 plants are fully fertile and develop viable gametophytes (Fig. 2, D and E; Supplemental Figs. S3, C and D, and S4, A and B). These results demonstrate that the functionality of the *IDA* gene in Col-0 plants is not linked to the observed gametophyte abortion phenotypes in *ida-2* outcrosses. Additionally, the genome of the new *idaCR* mutant line was sequenced and no additional T-DNA insertions were found, making *idaCR* a safe and effective replacement for *ida-2*. Additional information can be found in the supplementary information of this article which contains the Materials and methods and lists of primers (Supplemental Table S5). The raw sequencing data are available in the European Nucleotide Archive (ENA) repository (PRJEB62448).

**Figure 2.**
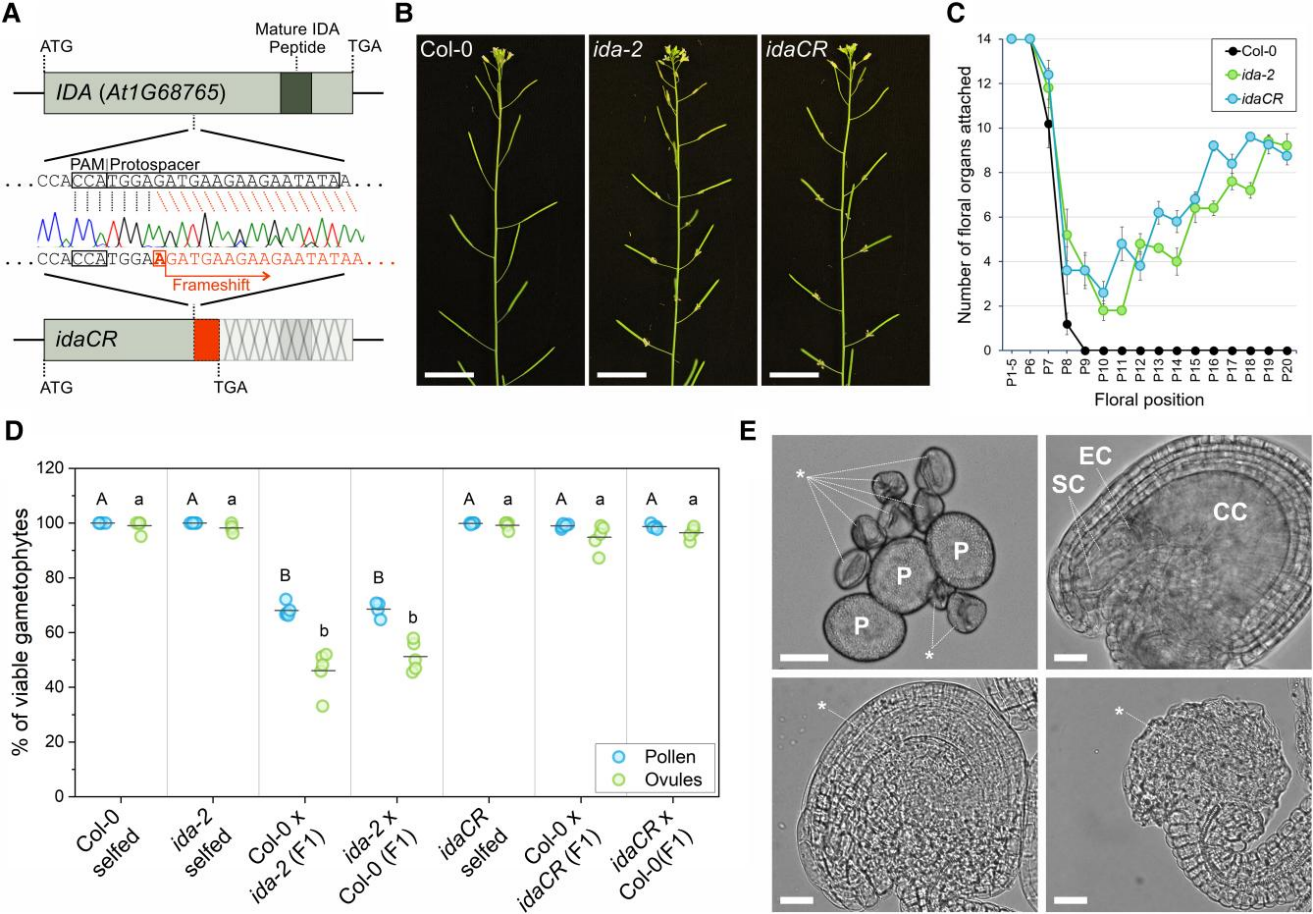
The new *idaCR* allele is an effective alternative to *ida-2*. **A)** Diagram of the *IDA* WT gene and the *idaCR* allele. The light gray box highlights the *IDA* coding sequence, while the dark gray box indicates the stretch of nucleotides that encodes the mature and active IDA peptide. Underneath, the protospacer and protospacer-adjacent motif (PAM) sequences used to generate *idaCR* with CRISPR-Cas9 are shown. Below, the 1-nucleotide insertion and resulting frameshift in the *idaCR* line are represented. On the bottom, the resulting *idaCR* model is shown. After the 1-nucleotide insertion site, a frameshift includes a small tract of amino acids until a stop codon is reached. The rest of the *IDA* gene after the frameshift is therefore not translated, including the mature IDA peptide. **B)** Representative images of floral organ abscission phenotypes in the *ida-2* and *idaCR* lines. Scale bars are 2 cm. **C)** Quantification of the floral organ abscission defect in *ida-2* and *idaCR* plants. Five plants per line were analyzed. The graph shows the mean ± Se of floral organs attached to the flower at each floral position. **D)** Quantification of viable male and female gametophytes in stage 15 flowers of F1 plants from selfed Col-0, *ida-2*, *idaCR*, and their reciprocal crosses. Different letters indicate statistically significant differences in a 1-way ANOVA with post hoc pairwise comparisons using Bonferroni and Tukey tests (*P* < 0.05). Five plants per genotype were analyzed, averaging the number of pollen grains belonging to each category from 4 flowers per plant. Counts per flower ranged between 50 and 250 pollen grains. Dissected ovules from 3 flowers per plant were analyzed and assigned to viable or aborted categories based on their morphology, ranging between 36 and 180 ovules per plant. **E)** Representative images of viable and aborted gametophytes. Top left, pollen grains from F1 plants of the cross between *ida-2* and Col-0 are shown. *P* corresponds to viable pollen grains; * marks aborted gametophytes. Top right, a viable and unfertilized ovule from an *ida-2* plant is shown. SC, synergid cells; EC, egg cell; CC, central cell. The 2 images below show the most encountered aborted ovule phenotypes in the F1 generation of the cross between *ida-2* and Col-0. We speculate they may correspond to early and late ovule abortion phenotypes (left and right, respectively). Scale bars are 20 *µ*m.

The mechanisms behind T-DNA integration and T-DNA-induced genomic rearrangements are not well understood. Different models have been proposed to explain T-DNA integration in the plant genome. T-DNAs may preferentially integrate at double-strand breaks in the DNA at random locations in the plant genome, and/or they may integrate at genomic sites with microhomology to the T-DNA (Bundock and Hooykaas 1996; Salomon and Puchta 1998; Tzfira et al. 2004; Kleinboelting et al. 2015; Zhang et al. 2018). Multiple DNA repair mechanisms have been suggested to influence or mediate T-DNA integration, but it is still unclear how this process takes place and which of them are actively mediating the integration (for review, see (Singer 2018; Gelvin 2021)). Experimental evidence supports the connection between double-strand DNA break repair mechanisms and the occurrence of T-DNA-induced translocations, inversions, deletions, or duplications in the plant genome (Hu et al. 2017). Under the model proposed by Hu et al., each edge of a single T-DNA would be incorporated onto a double-strand break in 2 independent chromosomes leading to a chromosomal translocation. Independently of the mechanism behind it, genomic structural variation is often a consequence of the Agrobacterium T-DNA transfer process, found in frequencies of up to 19% in some of the most used T-DNA collections (Clark and Krysan 2010; Pucker et al. 2021). The negative impact of genomic structural variation on gametophyte development and fertility of hybrids has been long noticed (Burnham 1930). In brief, when cells from a hybrid containing 2 structurally diverse versions of the same genome undergo meiosis, the complementarity between large portions of unrelated chromosomes results in the formation of multivalent chromosomal structures (Curtis et al. 2009; Valente et al. 2018). When meiosis resolves, such multivalent structures can yield gametophytes containing double doses of certain genomic regions as well as null doses of others, directly impairing the gametophyte's capacity to continue developing and/or reproduce (see (Curtis et al. 2009) for detailed models and alternatives).

As we have shown, T-DNA-induced chromosomal translocations in the *ida-2* line can result in problematic and unnecessary experimental uncertainty in genetic and developmental studies. We hope our findings will prove useful in warning the community on the use of the *ida-2* line and welcome the *idaCR* mutant as a safer alternative to future genetic work in abscission and/or peptide signaling studies. Seeds of the *idaCR* line have been deposited in the Nottingham Arabidopsis Stock Centre (NASC) to facilitate their distribution (NASC identifier: N2111653).

## Supplementary Material

kiad449_Supplementary_Data

## Data Availability

The data that support the findings of this study are available as indicated in the main text of the manuscript. For additional enquiries please contact the corresponding author.

## Supplemental data

The following materials are available in the online version of this article.

**Supplemental Figure S1**. Double hemizygous plants in the F2 generation of an *ida-2* × *idl1cr1* cross display reduced seed set and high proportions of developmentally impaired gametophytes.

**Supplemental Figure S2**. The reduction in seed set per silique in *ida-2* selfed plants is not caused by the disruption of the *IDA* gene or could be ecotype-dependent.

**Supplemental Figure S3.** Additional information to main Fig. 2.

**Supplemental Figure S4.** Additional information to main Fig. 2.

**Supplemental Table S1.** Genotyping of the F2 generation of an *ida-2* × *idl1cr1* genetic cross.

**Supplemental Table S2.** Presence of each of the 11 loci genotyped in 92 F2 plants of the *ida-2* × *idl1cr1* cross.

**Supplemental Table S3**. Presence of plants with each of the 3 expected genotypes for every mutation genotyped in 92 F2 plants of the cross between *ida-2* and *idl1cr1*.

**Supplemental Table S4**. Pairwise segregation analysis for the genotyped mutations from different chromosomes in the 92 F2 plants of the cross between *ida-2* and *idl1cr1*.

**Supplemental Table S5**. Primers used in this study.

## Dive Curated Terms

The following phenotypic, genotypic, and functional terms are of significance to the work described in this paper:

AT3G16555 Gramene: AT3G16555
AT3G16555 Araport: AT3G16555

AT5G18550 Gramene: AT5G18550
AT5G18550 Araport: AT5G18550

IDL1 Gramene: AT3G25655
IDL1 Araport: AT3G25655

IDL4 Gramene: AT3G18715
IDL4 Araport: AT3G18715

IDA Gramene: AT1G68765
IDA Araport: AT1G68765

AT3G20490 Gramene: AT3G20490
AT3G20490 Araport: AT3G20490

AT3G25655 Gramene: AT3G25655
AT3G25655 Araport: AT3G25655
