# Supplementary material for: Reproductive defects in the abscission mutant ida-2 are caused by T-DNA–induced genomic rearrangements
Source: Plant Physiol. 2023 Aug 9;193(4):2292–7. doi: 10.1093/plphys/kiad449 (PMC10663105; doi:10.1093/plphys/kiad449)
Supplement: kiad449_Supplementary_Data [file kiad449_supplementary_data.pdf]

Supplemental Figures, Tables and Materials and Methods to:

Alling & Galindo-Trigo. Reproductive defects in the abscission mutant *ida-2* are caused by T-DNA induced genomic rearrangements

Supplemental Figures

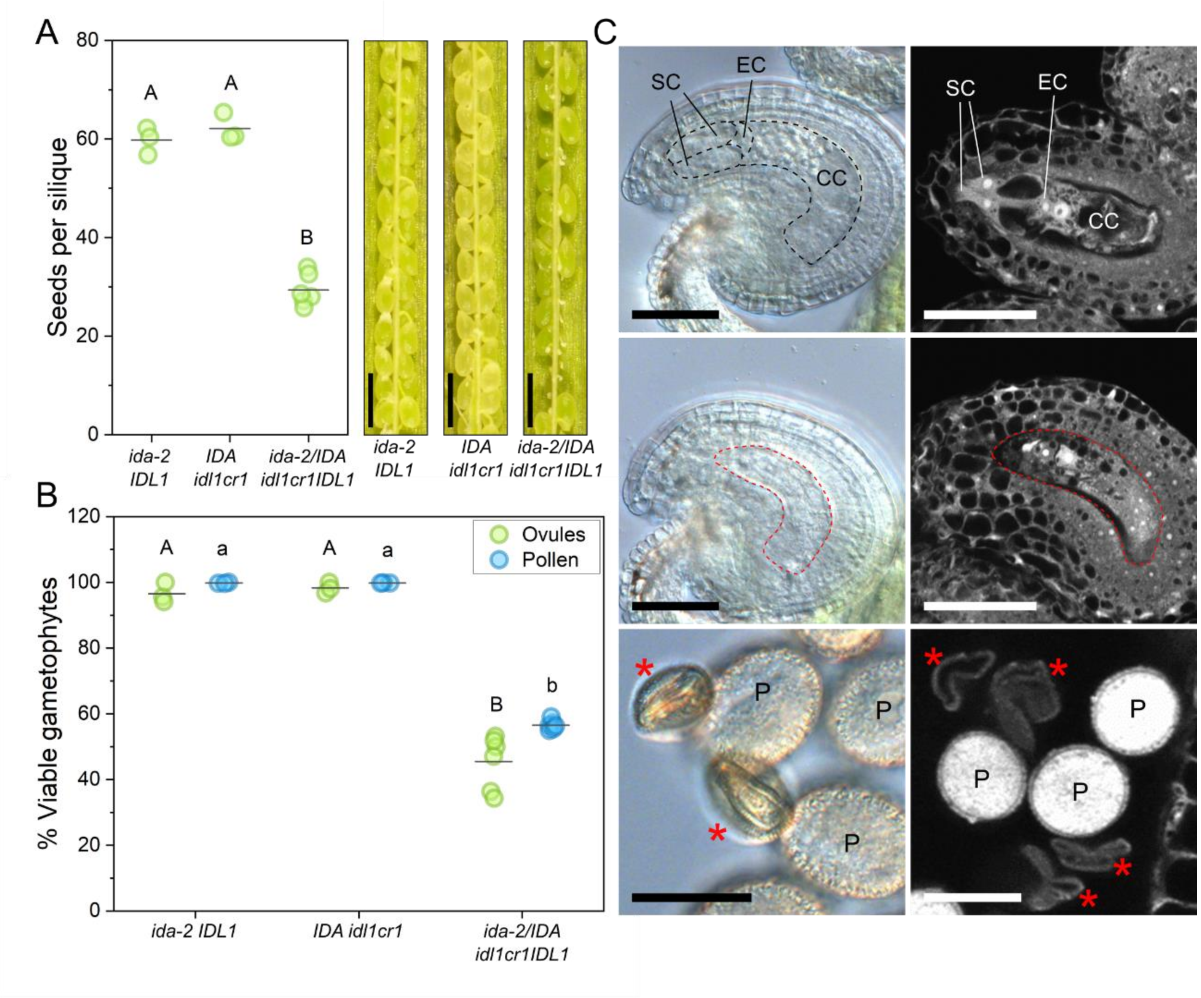

Supplemental Figure S1. Double hemizygous plants in the F2 generation of an *ida-2* x *idl1cr1* cross display reduced seed set and high proportions of developmentally impaired gametophytes. A, quantification of seeds per silique and representative images of dissected siliques. Three to six independent F2 plants corresponding to each genotype were selected, with five siliques per plant dissected and scored. **B**, percentage of viable gametophytes in non-fertilized stage 15 flowers from the same set of plants as A. Between 34 and 68 ovules and between 320 and 1726 pollen grains were quantified per plant, using three independent flowers per plant. **C**, representative images showing the morphological features of viable and aborted gametophytes as quantified on panel B. To the left, widefield microscope images of live ovules and pollen are shown. The right column contains confocal micrographs of fixed ovules and pollen grains using Christensen's fixative. The first row shows healthy female gametophytes, and the letters highlight the Central Cell (CC), Egg Cell (EC), and Synergid Cells (SC). The middle row exemplifies the aborted female gametophyte phenotype in which none of the mature female gametophyte cells can be observed. On the bottom row, viable (P) and aborted (*) pollen grains can be seen. At this stage, aborted pollen grains (*) appear to have dried and are devoid of cytoplasmic contents. Scale bars are 0.5 mm in the dissected silique photographs; 25 and 20 µm for ovule and pollen micrographs, respectively. Different letters indicate statistically significant differences in a one-way ANOVA with post hoc pairwise comparisons using Bonferroni and Tukey tests ($p < 0.05$).

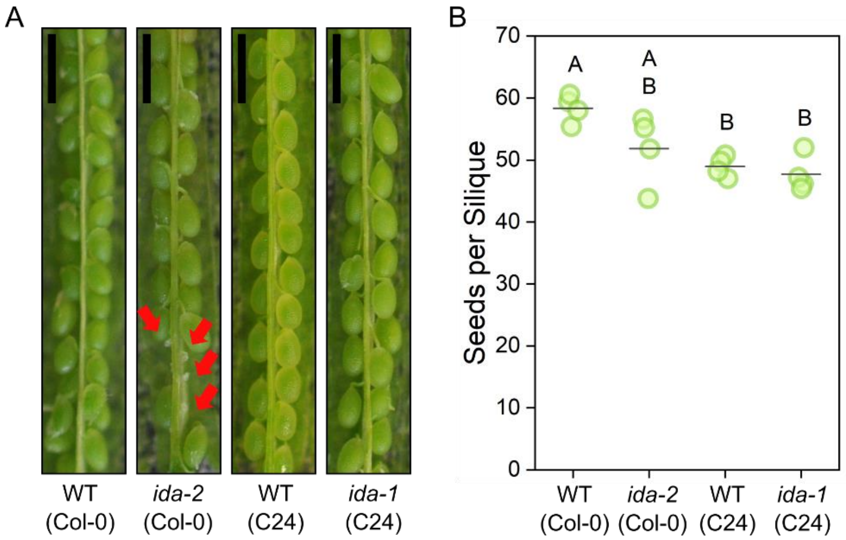

Supplemental Figure S2. The reduction in seed set per silique in *ida-2* selfed plants is not caused by the disruption of the *IDA* gene or could be ecotype-dependent. A, representative photographs of dissected siliques from selfed plants of the indicated genotypes. Red arrows indicate spurious seed positions that do not develop into mature seeds in the *ida-2* line Scale bars are 0.5 mm. **B**, quantification of the seeds per silique in four plants per genotype, counting seeds from five silique per plant. Different letters indicate statistically significant differences in a one-way ANOVA with post hoc pairwise comparisons using Bonferroni and Tukey tests ($p < 0.05$). As per Figure 1B, the seed set in *ida-2* plants is slightly reduced in comparison to its wild-type counterpart (Col-0). An *ida* T-DNA allele in the C24 accession background, *ida-1*, does not produce less seeds per silique than C24 wildtype. As an additional note, this decrease in seed production in *ida-2* plants is observed most frequently in the first 10-15 siliques produced by the main inflorescence; it is however very variable between siliques from the same plant, and between different batches of plants grown at different times (See also Figure S3). We hypothesize it could be due to suboptimal self-pollination, although this hypothesis remains to be tested.

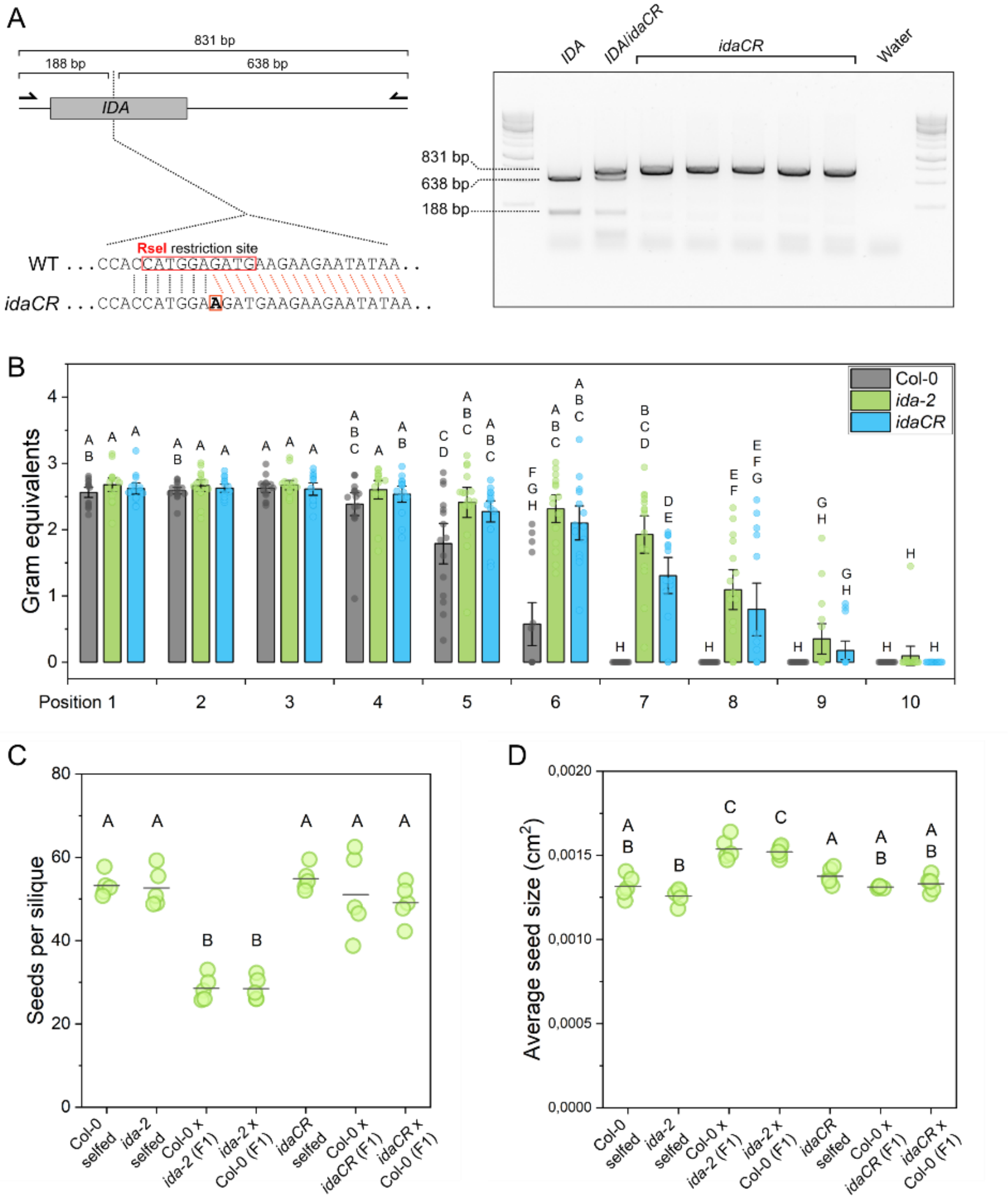

Supplemental Figure S3. Additional information to main Figure 2. A, genotyping strategy of the *idaCR* allele showing a diagram of the genotyping PCR and restriction digest with the enzyme RseI and an example of agarose electrophoresis gel containing the result of the genotyping of a wild-type plant (*IDA*), a hemizygous plant (*IDA*/*idaCR*), five homozygous *idaCR* plants, and a water sample control. **B**, the force required to detach petals from the floral positions indicated in the x axis. Both *ida-2* and *idaCR* plants have more strongly attached petals than wild-type Col-0 at positions 6, 7 and 8 as a consequence of the abscission impairment in these mutants. Different letters indicate statistically significant differences in a two-way ANOVA with post hoc pairwise comparisons using Bonferroni and Tukey tests ($p < 0.05$). Between 14 and 15 plants per genotype were analyzed, and between 1 and 3 petals measured and averaged per flower and plant. Bars represent the mean and whiskers are ± 1.5 SE. **C**, quantification of the average mature seed production per silique in the F1 generation of the crosses indicated in the x axis. The decrease in seed production per silique in the *ida-2* crosses to wild-type is

the consequence of female gametophyte abortion at high rates shown in Figure 2. See also Figure S4A. **D**, quantification of average seed size in the same genotypes as shown in C. Seeds growing in the ovaries of the *ida-2* crosses with wild-type are significantly larger than wild-type, as expected of seeds grown in siliques where many ovules are not fertilized. In panels C and D, different letters indicate statistically significant differences in a one-way ANOVA with post hoc pairwise comparisons using Bonferroni and Tukey tests ($p < 0.05$). Five plants per genotype were analyzed, averaging the number/size of seeds from four siliques per plant.

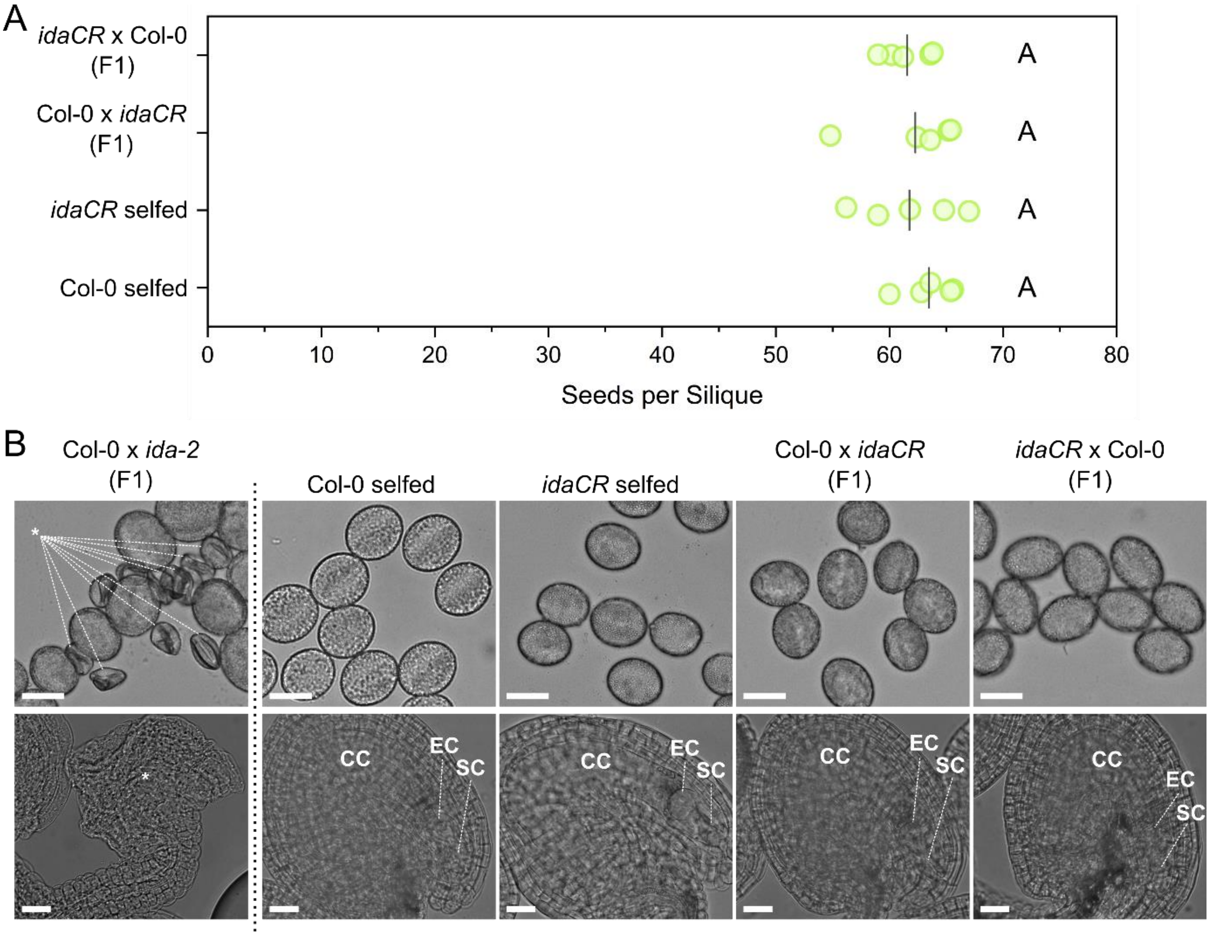

Supplemental Figure S4. Additional information to main Figure 2. A, quantification of the average seed production per silique in the F1 generation of the crosses between Col-0 and *idaCR*. This panel contains an additional replicate of the quantification shown in Figure S3C in which no statistically significant differences were detected either. Statistically significant differences tested in a one-way ANOVA with post hoc pairwise comparisons using Bonferroni and Tukey tests ($p < 0.05$). Five plants per genotype were analyzed, averaging the number of seeds from four siliques per plant. **B**, representative images of the crosses between Col-0 and *idaCR* showing indistinguishable male and female gametophyte morphologies. For comparison, an example of the pollen and ovule abortion phenotypes of *ida-2* outcrosses are shown on the lefthand side (marked with a white *). SC, synergid cells; EC, egg cell; CC, central cell. Scale bars are 20 µm.

Supplemental Tables

Supplemental Table S1. Genotyping of the F2 generation of an *ida-2* x *idl1cr1* genetic cross. The table shows the number of plants recovered with each of the expected genotype combinations in the F2 population of the *ida-2* x *idl1cr1* cross. Also shown the expected proportions of plants for each category and the obtained p-value in a Chi square test comparing both groups (F2 plants vs Expected), indicating a significant difference between observed and expected number of plants.

Genotype	Plants	Expected	ChiSq Test
IDA/IDA IDL1/IDL1	0	6,3125	3,40553E-39
IDA/IDA idl1cr1/IDL1	2	12,625	
IDA/IDA idl1cr1/idl1cr1	27	6,3125	
ida-2/IDA IDL1/IDL1	0	12,625	
ida-2/IDA idl1cr1/IDL1	47	25,25	
ida-2/IDA idl1cr1/idl1cr1	0	12,625	
ida-2/ida-2 IDL1/IDL1	25	6,3125	
ida-2/ida-2 idl1cr1/IDL1	0	12,625	
ida-2/ida-2 idl1cr1/idl1cr1	0	6,3125	
**Total**	101	101	

Supplemental Table S2. Presence of each of the eleven loci genotyped in 92 F2 plants of the *ida-2* x *idl1cr1* cross. A Chi square test against the expected presence indicates all loci are being transmitted as expected in an F2 population of a cross between homozygous plants for each allele ($p > 0.05$).

Genotyped locus	Detected	Expected
IDA WT locus	64	69
IDA TDNA Start	69	69
IDA TDNA End	68	69
IDL1 WT locus	69	69
idl1cr1	66	69
KNO1 WT locus	66	69
KNO1 TDNA Start	69	69
KNO1 TDNA End	68	69
AT5G18550 WT locus	73	69
AT5G18550 TDNA Start	66	69
AT5G18550 TDNA End	66	69
	**Chi Square**	0,999680967

Supplemental Table S3. Presence of plants with each of the three expected genotypes for every mutation genotyped in 92 F2 plants of the cross between *ida-2* and *idl1cr1*. When individually tested, none of the mutations have impaired transmission based on a Chi square test comparing the detected and expected number of plants corresponding to each genotype category.

A. Segregation of Chr1 T-DNA insertion

Gene	Total	Expected	Chi Square
IDA/IDA	23	23	0,442543112
IDA/ida-2	41	46	
ida-2/ida-2	28	23	

B. Segregation of Chr3 T-DNA insertion

Gene	Total	Expected	Chi Square
KNO1/KNO1	22	23	0,770381398
KNO1/kno1	44	46	
kno1/kno1	26	23	

C. Segregation of Chr5 T-DNA insertion

Gene	Total	Expected	Chi Square
AT5G18550/AT5G18550	26	23	0,574446778
AT5G18550/at5g18550	47	46	
at5g18550/at5g18550	19	23	

D. Segregation of *idl1cr1* mutation

Gene	Total	Expected	Chi Square
IDL1/IDL1	26	23	0,745665433
IDL1/idl1cr1	43	46	
idl1cr1/idl1cr1	23	23	

Supplemental Table S4. Pairwise segregation analysis for the genotyped mutations from different chromosomes in the 92 F2 plants of the cross between *ida-2* and *idl1cr1*. A and B, unexpected segregation ratios are observed when considering *IDA* and both mutations in chr3 (*IDL1* and *KNO1*). Segregation ratios are strongly skewed towards the original parental genotypes (highlighted in red). **C, D and E**, the mutation in chr5 (*AT5G18550*), however, segregates as expected from a genomic locus located in a different chromosome from *IDA*, *IDL1*, and *KNO1*. Chi square tests comparing the detected and expected number of plants corresponding to each genotype category are shown.

A. F2 distribution of *IDA* and *IDL1* mutations

Gene1	Gene2	Total	Expected	Chi Sq
IDA/IDA	*IDL1/IDL1*	0	5,75	**3,31E-32**
IDA/IDA	*IDL1/idl1cr*	1	11,5	
IDA/IDA	*idl1cr/idl1cr*	22	5,75	
IDA/ida	*IDL1/IDL1*	1	11,5	
IDA/ida	*IDL1/idl1cr*	39	23	
IDA/ida	*idl1cr/idl1cr*	1	11,5	
ida/ida	*IDL1/IDL1*	25	5,75	
ida/ida	*IDL1/idl1cr*	3	11,5	
ida/ida	*idl1cr/idl1cr*	0	5,75	

B. F2 distribution of *IDA* and *KNO1* mutations

Gene1	Gene2	Total	Expected	Chi Sq
IDA/IDA	*KNO1/KNO1*	22	5,75	**1,12E-34**
IDA/IDA	*KNO1/kno1*	1	11,5	
IDA/IDA	*kno1/kno1*	0	5,75	
IDA/ida	*KNO1/KNO1*	1	11,5	
IDA/ida	*KNO1/kno1*	40	23	
IDA/ida	*kno1/kno1*	0	11,5	
ida/ida	*KNO1/KNO1*	0	5,75	
ida/ida	*KNO1/kno1*	2	11,5	
ida/ida	*kno1/kno1*	26	5,75	

C. F2 distribution of *IDL1* and *AT5G18550* mutations

Gene1	Gene2	Total	Expected	Chi Sq
IDL1/IDL1	*AT5G18550/AT5G18550*	4	5,75	0,420834
IDL1/IDL1	*AT5G18550/at5g18550*	16	11,5	
IDL1/IDL1	*at5g18550/at5g18550*	6	5,75	
IDL1/idl1cr	*AT5G18550/AT5G18550*	11	11,5	
IDL1/idl1cr	*AT5G18550/at5g18550*	21	23	
IDL1/idl1cr	*at5g18550/at5g18550*	11	11,5	
idl1cr/idl1cr	*AT5G18550/AT5G18550*	10	5,75	
idl1cr/idl1cr	*AT5G18550/at5g18550*	11	11,5	
idl1cr/idl1cr	*at5g18550/at5g18550*	2	5,75	

D. F2 distribution of *IDA* and *AT5G18550* mutations

Gene1	Gene2	Total	Expected	Chi Sq
IDA/IDA	*AT5G18550/AT5G18550*	11	5,75	0,214324
IDA/IDA	*AT5G18550/at5g18550*	10	11,5	

IDA/IDA	*at5g18550/at5g18550*	2	5,75	
IDA/ida	*AT5G18550/AT5G18550*	10	11,5	
IDA/ida	*AT5G18550/at5g18550*	20	23	
IDA/ida	*at5g18550/at5g18550*	11	11,5	
ida/ida	*AT5G18550/AT5G18550*	5	5,75	
ida/ida	*AT5G18550/at5g18550*	17	11,5	
ida/ida	*at5g18550/at5g18550*	6	5,75	

E. F2 distribution of *KNO1* and *AT5G18550* mutations

Gene1	Gene2	Total	Expected	Chi Sq
KNO1/KNO1	*AT5G18550/AT5G18550*	11	5,75	0,268091
KNO1/KNO1	*AT5G18550/at5g18550*	10	11,5	
KNO1/KNO1	*at5g18550/at5g18550*	2	5,75	
KNO1/kno1	*AT5G18550/AT5G18550*	11	11,5	
KNO1/kno1	*AT5G18550/at5g18550*	21	23	
KNO1/kno1	*at5g18550/at5g18550*	11	11,5	
kno1/kno1	*AT5G18550/AT5G18550*	4	5,75	
kno1/kno1	*AT5G18550/at5g18550*	16	11,5	
kno1/kno1	*at5g18550/at5g18550*	6	5,75	

Supplemental Table S5. Primers used in this study.

Routine genotyping primer sequences

Purpose	Forward Primer Sequence	Reverse Primer Sequence
ida-2 (WT allele)	GATCTTTATATGGCCGTAATGACC	TTCATTCATAAGACCCTTCATTCA
ida-2 (T-DNA allele)	TTCATTCATAAGACCCTTCATTCA	ATTTTGCCGATTCGGAAC
idl1cr1 (PCR + BslI digest)	GTAATCACACGTACGTACACCATCG	TTATGTCTCATGGAAGGTCCCGAAG
idaCR (PCR + RseI digest)	TTCATTCATAAGACCCTTCATTCA	TGGGCATTTGGTCTAGTGGT

Segregation analysis of multiple T-DNA insertions found in the *ida-2* T-DNA line

Purpose	Forward Primer Sequence	Reverse Primer Sequence
IDA WT allele	GATCTTTATATGGCCGTAATGACC	TTCATTCATAAGACCCTTCATTCA
IDA - T-DNA junction 1	GATCTTTATATGGCCGTAATGACC	ATTTTGCCGATTCGGAAC
IDA - T-DNA junction 2	TTCATTCATAAGACCCTTCATTCA	ATTTTGCCGATTCGGAAC
KNO1 WT allele	CCTGATGCAAACCCTCTTCTAT	TAACCAGCCTTCAGAATTGTGT
KNO1 - T-DNA junction 1	TAACCAGCCTTCAGAATTGTGT	ATTTTGCCGATTCGGAAC
KNO1 - T-DNA junction 2	CCTGATGCAAACCCTCTTCTAT	ATTTTGCCGATTCGGAAC
AT5G18550 WT allele	CTGTTACTCCTGTCTCCCTA	ATCTGCAAGAGGTTCCGAATTTA
AT5G18550 - T-DNA junction 1	CTGTTACTCCTGTCTCCCTA	ATTTTGCCGATTCGGAAC
AT5G18550 - T-DNA junction 2	AAAACCAACCCCAGTACATT	CAATCTCTGCGGTTCTCATA

Cloning primers to generate the *idaCR* mutant

Purpose	Forward Primer Sequence	Reverse Primer Sequence
Introduction of protospacer sequence targeting *IDA* in the vector *pKI1.1R*	ATTG ATATTCTTCTTCATCTCCA	AAAC TGGAGATGAAGAAGAATAT

Supplemental Materials and Methods

Growth conditions and plant materials

Seeds were germinated on MS plates containing 0.7% sucrose and seedlings transferred to soil on the seventh day. Plates and plants on soil were grown in environmentally controlled conditions with long photoperiod (16 h of light and 8 h of darkness), constant temperature of 22ºC and light intensity of approximately 150 µmol/m^2. The lines used as controls in this study are Col-0 and C24. Mutant lines used are *ida-2* (Col-0 accession background; SALK_133209; (Cho et al., 2008)), *idl1cr1* (Col-0 accession background; (Shi et al., 2018)) and *ida-1* (C24 accession background; (Butenko et al., 2003)). T-DNA insertions were genotyped with Taq DNA Polymerase (NEB), whereas single-nucleotide polymorphisms (SNPs) present in the gene-edited lines were detected by PCR amplification with Q5 High Fidelity PCR Polymerase (NEB) followed by restriction digest with the corresponding restriction enzyme (see supplementary Table S5 for primer sequences and restriction enzymes).

Generation of the ida-CRISPR (idaCR) line

A protospacer with sequence TGGAGATGAAGAAGAATATA targeting the *IDA* gene was selected using CRISPR-P 2.0 (Liu et al., 2017). The protospacer sequence was cloned into the genome editing expression vector *pKAMA-ITACHI1.1R* via AarI digestion/ligation. Col-0 wild-type plants transformed with the *pKAMA-ITACHI1.1R-IDA* construct by the floral dip method (Clough and Bent, 1998; Tsutsui and Higashiyama, 2017). T1 transformants were selected on hygromycin-containing MS plates and the disruption of the *IDA* gene checked by genotyping and sanger sequencing of the PCR-amplified genomic region (See Figure S4A and Table S5). A transformant with a homozygous 1-nucleotide insertion in the 34th codon of *IDA* was selected and backcrossed to Col-0 to remove the CRISPR-Cas9 T-DNA insertion from its background. A descendant with the homozygous *idaCR* 1-nucleotide insertion that lacked the CRISPR-Cas9 T-DNA insertion was selected by genotyping, sensitivity to hygromycin as well as absence of seed red fluorescence (both selection markers present in the *pKAMA-ITACHI1.1R* vector).

Phenotyping assays

To observe the female gametophyte structure in live ovules, stage 15 flowers were emasculated and dissected after an additional 24h in the growth chamber (results shown in Figure S1); alternatively, stage 13 flowers were emasculated and kept in the growth chamber for 48-72h prior to dissection and microscopy (results shown in Figure 2 and S3). Dissected ovules were mounted on half-strength MS with 5% sucrose and immediately imaged on a widefield microscope with differential contrast interference. Mature pollen grains were collected from freshly dehisced anthers in stage 15 flowers and mounted on half-strength MS with 5% sucrose for imaging in a widefield microscope. Confocal imaging of female and male gametophytes was conducted following the protocol by Christensen and colleagues (Christensen et al., 1997).

Seed production per silique was quantified in green, fully elongated siliques by manually dissecting the siliques on double-sided sticky tape (Data shown in Figure 1, S1 and S2). Alternatively, mature dry siliques were individually harvested, their seeds imaged on a 1.5 x 2.5 cm grid using a Leica Z16apoA microscope connected to a Nikon D90 camera, with number of seeds and size estimated with ImageJ using the “Threshold” and “Analyze particles” functions (https://imagej.nih.gov/ij/; data presented in Figure S3).

The floral organ abscission impairment in *ida-2* and *idaCR* lines was quantified by counting the floral organs attached to the flowers in the main inflorescence. In brief, the main inflorescence stem was cut from the plant, gently shaken four times, and the number of floral organs that remained attached to each floral position (P1-P20) was recorded by visual inspection. The floral organs in the *ida-2* and *idaCR* lines are very weakly attached to the flower from position 8 to position 12, after which it is increasingly

common to find strongly attached floral organs. The floral organ retention profile faithfully mirrors the petal break-strength (PBS, also known as petal break-force) profile previously shown for the *ida-2* genotype (Liu et al., 2013). Additionally, the abscission phenotype in terms of PBS gram equivalents was quantified in one to three petals per floral position from the main inflorescence of each plant with a force transducer as per (Stenvik et al., 2008).

Whole-genome sequencing and genomic analysis

High molecular weight DNA was extracted from Col-0, *ida-2* and *idaCR* plants for subsequent whole-genome sequencing using the PacBio HiFi technology. Briefly, between 1-2g of unopened flower buds from ~20 plants were harvested per genotype, nuclei were purified, and high molecular weight DNA extracted from the nuclei purification with the Circulomics Nanobind Plant Nuclei Big DNA Kit according to the manufacturer's instructions (Workman et al., 2018). Library was prepared following Pacific Biosciences protocol for HiFi library prep using SMRTbell Express Template Prep Kit 2.0, fragmenting the DNA to 15-20 kb fragments using Megaruptor 3 and applying a library size cutoff of 10kb with BluePippin. Libraries were pooled and sequenced on 8M SMRT cells using a Sequell II instrument and Sequel II Binding kit 2.2 with Sequencing chemistry v2.0. HiFi reads were demultiplexed and assembled using the Genome Assembly pipeline SMRT Link v11.0.0.146107 with default settings, yielding 0.134/0.131/0.129 Gb assembled genome sizes and 21.94/14.66/12.02 HiFi read coverages for the Col-0/*ida-2*/*idaCR* lines, respectively (see additional details on the Supplementary files). Sequencing data can be freely accessed in the ENA repository under the project PRJEB62448.

The T-DNA insertions in the *ida-2* line were initially analyzed with "loreta" inputting the HiFi reads we recovered (Pucker et al., 2021). The tool was successful in identifying the T-DNA – chromosome junctions in chromosome 1 (*IDA*), chromosome 3 (*KNO1*, *AT3G20490*) and chromosome 5 (*AT5G18550*). However, loreta's outputs contained various alternative predictions for each of the T-DNAs supported by reads with similar confidence. These T-DNA structural predictions were deemed not biologically relevant upon examination of the reads used to support such arrangements, and given the long and repetitive nature of the T-DNA insertions and an average HiFi read length of approximately 14 kb that was unable to bridge both junctions of the T-DNA insertion. We therefore chose to use genotyping of a segregating F2 population as the strategy to understand the structure of the genome surrounding each T-DNA insertion. Next we identified the T-DNA-containing sequences with BLASTn using the *pROK2* vector sequence as query (Altschul et al., 1990). A BLAST database was created with the T-DNA-containing HiFi reads and was used to recover the set of reads that contained each of the chromosome-T-DNA junctions. Each of the junction read sets were independently aligned using Clustal Omega and manually curated to remove small gaps and generate the consensus sequence used to produce the maps shown in Figure 1C (Sievers et al., 2011). Assembled chromosome – T-DNA junction sequences can be found in the supplementary files. In parallel, this pipeline was applied to the *idaCR* genomic data using the *pKAMA-ITACHI1.1R-IDA* plasmid sequence to identify possible undetected insertions in the backcrossed idaCR line. The loreta pipeline did not identify T-DNA insertions in *idaCR*. Additionally, no *pKAMA-ITACHI1.1R-IDA* – containing reads were found with BLASTn, indicating that the *idaCR* line is free from transgenic genomic sequences. Additionally, the genomic sequences were subjected to SNP and small insertion and deletion (INDEL) analysis. PacBio HiFi reads were mapped to the TAIR10 reference genome using Minimap2 with default setting for PacBio/Oxford Nanopore sequencing data (Li, 2018). Mapped reads from *ida-2*, *idaCR* and Col-0 were merged and SNP/INDEL variants were called with freebayes (Garrison and Marth, 2012). Variants were processed and filtered with VcfAllelicPrimitives, SnpEff and SnpSift (Cingolani et al., 2012; Garrison et al., 2022). The list of detected homozygous variants with a non-synonymous effect on genes of *idaCR* and *ida-2* (with Col-0 as reference) can be found in the supplementary files, together with the full set of variants detected between Col-0 and the TAIR10 reference genome. Most of these analyses were run

with cloud-based Galaxy, while manual curation and visualization of results was performed with IGB and MegaX (Freese et al., 2016; Tamura et al., 2021; Galaxy, 2022).

Supplemental References

Altschul SF, Gish W, Miller W, Myers EW, Lipman DJ (1990) Basic local alignment search tool. J Mol Biol **215:** 403-410

Butenko MA, Patterson SE, Grini PE, Stenvik GE, Amundsen SS, Mandal A, Aalen RB (2003) Inflorescence deficient in abscission controls floral organ abscission in Arabidopsis and identifies a novel family of putative ligands in plants. Plant Cell **15:** 2296-2307

Cho SK, Larue CT, Chevalier D, Wang HC, Jinn TL, Zhang SQ, Walker JC (2008) Regulation of floral organ abscission in Arabidopsis thaliana. Proceedings of the National Academy of Sciences of the United States of America **105:** 15629-15634

Christensen CA, King EJ, Jordan JR, Drews GN (1997) Megagametogenesis in Arabidopsis wild type and the Gf mutant. Sexual Plant Reproduction **10:** 49-64

Cingolani P, Platts A, Wang le L, Coon M, Nguyen T, Wang L, Land SJ, Lu X, Ruden DM (2012) A program for annotating and predicting the effects of single nucleotide polymorphisms, SnpEff: SNPs in the genome of Drosophila melanogaster strain w1118; iso-2; iso-3. Fly (Austin) **6:** 80-92

Clough SJ, Bent AF (1998) Floral dip: a simplified method for Agrobacterium-mediated transformation of Arabidopsis thaliana. Plant J **16:** 735-743

Freese NH, Norris DC, Loraine AE (2016) Integrated genome browser: visual analytics platform for genomics. Bioinformatics **32:** 2089-2095

Galaxy C (2022) The Galaxy platform for accessible, reproducible and collaborative biomedical analyses: 2022 update. Nucleic Acids Res **50:** W345-W351

Garrison E, Kronenberg ZN, Dawson ET, Pedersen BS, Prins P (2022) A spectrum of free software tools for processing the VCF variant call format: vcflib, bio-vcf, cyvcf2, hts-nim and slivar. PLOS Computational Biology **18:** e1009123

Garrison E, Marth G (2012) Haplotype-based variant detection from short-read sequencing. arXiv preprint arXiv:1207.3907

Li H (2018) Minimap2: pairwise alignment for nucleotide sequences. Bioinformatics **34:** 3094-3100

Liu B, Butenko MA, Shi CL, Bolivar JL, Winge P, Stenvik GE, Vie AK, Leslie ME, Brembu T, Kristiansen W, Bones AM, Patterson SE, Liljegren SJ, Aalen RB (2013) NEVERSHED and INFLORESCENCE DEFICIENT IN ABSCISSION are differentially required for cell expansion and cell separation during floral organ abscission in Arabidopsis thaliana. Journal of Experimental Botany **64:** 5345-5357

Liu H, Ding Y, Zhou Y, Jin W, Xie K, Chen LL (2017) CRISPR-P 2.0: An Improved CRISPR-Cas9 Tool for Genome Editing in Plants. Mol Plant **10:** 530-532

Pucker B, Kleinbolting N, Weisshaar B (2021) Large scale genomic rearrangements in selected Arabidopsis thaliana T-DNA lines are caused by T-DNA insertion mutagenesis. Bmc Genomics **22**

Shi CL, von Wangenheim D, Herrmann U, Wildhagen M, Kulik I, Kopf A, Ishida T, Olsson V, Anker MK, Alberts M, Butenko MA, Felix G, Sawa S, Claassen M, Friml J, Aalen RB (2018) The dynamics of root cap sloughing in Arabidopsis is regulated by peptide signalling. Nature Plants **4:** 596-604

Sievers F, Wilm A, Dineen D, Gibson TJ, Karplus K, Li W, Lopez R, McWilliam H, Remmert M, Soding J, Thompson JD, Higgins DG (2011) Fast, scalable generation of high-quality protein multiple sequence alignments using Clustal Omega. Mol Syst Biol **7:** 539

Stenvik GE, Tandstad NM, Guo Y, Shi CL, Kristiansen W, Holmgren A, Clark SE, Aalen RB, Butenko MA (2008) The EPIP peptide of INFLORESCENCE DEFICIENT IN ABSCISSION is sufficient to induce abscission in Arabidopsis through the receptor-like kinases HAESA and HAESA-LIKE2. Plant Cell **20:** 1805-1817

Tamura K, Stecher G, Kumar S (2021) MEGA11: Molecular Evolutionary Genetics Analysis Version 11. Mol Biol Evol **38:** 3022-3027

Tsutsui H, Higashiyama T (2017) pKAMA-ITACHI Vectors for Highly Efficient CRISPR/Cas9-Mediated Gene Knockout in Arabidopsis thaliana. Plant Cell Physiol **58:** 46-56

Workman RE, Timp W, Fedak R, Kilburn D, Hao S, Liu KJ (2018) High Molecular Weight DNA Extraction from Recalcitrant Plant Species for Third Generation Sequencing. *In*,
